# High Efficiency and Long-Term Antibacterial Carbon Dots for Combating Antibiotic Resistance

**DOI:** 10.3390/nano15171296

**Published:** 2025-08-22

**Authors:** Beibei Wang, Dandan Zhang, Gang Zhou, Xiaodong Li, Tingli Sun, Qingshan Shi, Xiaobao Xie

**Affiliations:** 1 Guangdong Provincial Key Laboratory of Microbial Culture Collection and Application, State Key Laboratory of Applied Microbiology Southern China, Institute of Microbiology, Guangdong Academy of Sciences, Guangzhou 510070, China; wangbeibei@gdim.cn (B.W.);; 2 School of Life Sciences, Guangzhou University, Guangzhou 510006, China

**Keywords:** carbon dots, antibiotic resistance, antibacterial performance

## Abstract

Combating antibiotic resistance is critically significant for global public health. The development of new antibacterial nanomaterial is a promising way to do this. In this study, a bottom-up approach was employed to fabricate antibacterial carbon dots (ACDs). During the synthesis, quaternary ammonium function groups with long alkyl chains were successfully grafted on ACDs’ surfaces. The obtained ACDs exhibited potent inhibitory against methicillin-resistant *Staphylococcus aureus* (MRSA) bacteria with minimum inhibitory concentrations of 2.5 µg/mL. Crucially, 2.5 µg/mL of ACDs could inhibit the growth of MRSA for as long as 72 h, which highlighted their long-term activity. Mechanistic investigations revealed that ACDs exerted bactericidal effects for MRSA bacteria primarily through disrupting the cell wall/membrane, destroying cell membrane potential, inducing the generation of excessive ROS, and triggering the leakage of nucleic acids and intracellular components. In sum, this work provided a kind of ACD with high efficiency and long-term antibacterial activity, offering promising potential for combating drug-resistant bacterial infections.

## 1. Introduction

Bacterial infections, especially those caused by drug-resistant bacteria, are a serious public health problem worldwide [1,2]. Furthermore, the overuse and abuse of antibiotics has accelerated the emergence and evolution of drug-resistant bacteria [3,4,5]. On the one hand, many efforts have been made in rapid antimicrobial susceptibility testing technology, antimicrobial drug-resistant testing technology, and drug-resistant gene detecting technology [6,7,8,9]. These technologies help to combat antibiotic resistance. On the other hand, the development of novel materials and antibacterial ways to combat antibiotic resistance is urgent. Many nanomaterials, such as metal–organic frameworks, enzyme-loaded nanostructures, and photothermal materials, have been designed and applied for inactivating drug-resistant bacteria. As reported previously, zero-dimensional carbon-based nanomaterials, like fullerene, nanodiamonds, and carbon dots, have been shown to have both good antibacterial activity and biosafety [10,11,12]. Therefore, these carbon-based nanomaterials offer an effective alternative to eliminate drug-resistant bacterial infections.

As a novel type of zero-dimensional antibacterial nanomaterial, carbon dots (CDs) possess advantages such as good stability, eco-friendliness, and low cost, which have enabled them to be widely applied in the field of biomedicine [13,14,15]. In particularly, numerous studies on the antibacterial ability and antibacterial mechanisms of CDs have been carried out [16,17,18]. For instance, Du et al. synthesized a kind of CD derived from metformin using the electrochemical method and revealed that 200 µg/mL of CDs could achieve complete antibacterial activity against *Staphylococcus aureus* and *Escherichia coli* [19]. Biomass-derived CDs from mango peel have been applied in efficient bioimaging and antibacterial action [20], and CDs synthesized from curcumin and ethylenediamine exhibited broad-spectrum antibacterial ability against many types of pathogenic bacteria [21]. However, it is worth noting that while it is true that CDs have shown antimicrobial potential, limitations often depend on their surface functionalization, structure design, or target organism. The antibacterial efficiency of CDs varies depending on these parameters and the mechanistic understanding is still evolving for specific types of functionalized CDs.

In general, the unique structure of quaternary ammonium and phosphonium salts with long alkyl chains can enhance the antibacterial ability of materials by increasing hydrophobic interactions and penetration of cell membrane [22,23,24]. For example, Tan et al. found that the antibacterial and antifouling properties of cellulose acetate membrane were greatly improved via the surface grafting of quaternary ammonium salt [25]. In 2025, Wu et al. reported a kind of renewable rosin-based bis-quaternary ammonium salt with excellent antifungal activity [26]. Frolov et al. synthesized a variety of pyridinium-based quaternary ammonium compounds with a long alkyl part, and found the bis-quaternary ammonium biocide’s architectures showed improved antibacterial activity [27]. From the literature above, we found that the presence of pyridine and long alkyl chains can significantly enhance the antibacterial activity of the target product. Therefore, a hypothesis that CDs prepared by precursors containing pyridine and long alkyl chains would exhibit excellent antibacterial activity was proposed.

The general approaches to obtain CDs can be divided into top-down and bottom-up [28,29]. The top-down approach means breaking the bulk carbon materials (such as carbon nanotubes, graphene, graphite) into CDs through methods of ultrasonic peeling, arc discharge, laser ablation, etc. Due to shortcomings of time cost, complex operation, and poor quality of the product, this approach was always used in the early stage of CD preparation. The bottom-up approach means using small molecules, biomass, or drugs as starting materials to prepare CDs through treatment with the microwave method, hydrothermal method, or pyrolysis. During the synthesis process of the bottom-up approach, CDs could be prepared and functionalized in a “one-step” treatment. For this reason, the bottom-up approach has been widely applied in the preparation of CDs recently. However, it is worth noting that the methods of the bottom-up approach mentioned above usually require a high temperature, high pressure, or solvents with high boiling points during the synthesis process, which is not safe enough. Thus, a simpler and greener synthesis method for antibacterial carbon dots with quaternary ammonium groups urgently needs to be developed.

**Scheme 1:**
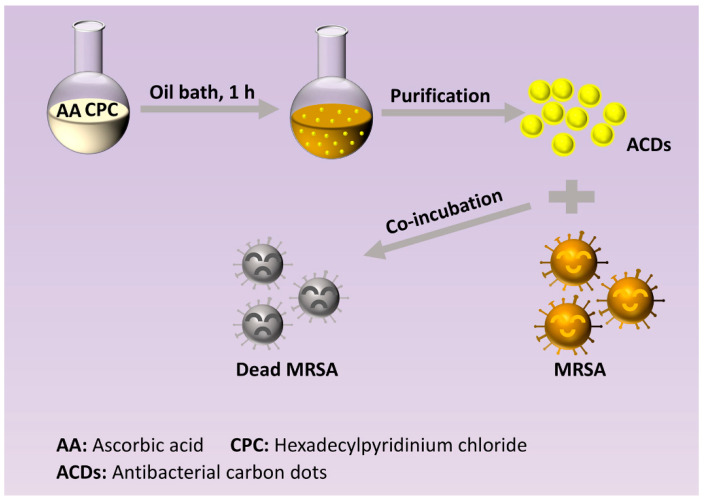
Synthesis route of ACDs and the application of ACDs for incubating bacteria.

Inspired by the studies above, in this work, an attempt was made to improve the antibacterial performance of CDs by the modification of quaternary ammonium groups under a mild condition. Antibacterial carbon dots (ACDs) were synthesized using ascorbic acid and hexadecylpyridinium chloride monohydrate via a bottom-up approach (Scheme 1). Herein, quaternary ammonium function groups with long alkyl chains were successfully grafted on the surface of CDs during their synthesis process. The obtained ACDs exhibited highly effective and long-term inhibition against MRSA bacteria. Crucially, a low concentration of ACDs could inhibit the growth of MRSA for as long as 72 h, which has rarely been reported. Notably, the characterization of bacterial morphology, live/dead staining analysis, cell membrane potential measurement, reaction oxygen species studies, and nucleic acids concentration studies were performed to reveal the underlying antibacterial mechanisms of ACDs. Thus, our work provided a kind of ACD with highly efficient and long-term antibacterial performance, which was promising for combating drug-resistant bacterial infections.

## 2. Experimental Section

### 2.1. Synthesis of ACDs

ACDs were prepared by a bottom-up method (Scheme 1). In brief, ascorbic acid (30 mmol) and hexadecylpyridinium chloride monohydrate (30 mmol) were dissolved into 80 mL sodium hydroxide solution (0.1 M), and subsequently heated at 80 °C for 1 h. After filtration with a 0.22 µm filter membrane and dialysis for 24 h, the resultant solution in the dialyzed bag was collected, and stored at room temperature for further use.

### 2.2. Antibacterial Assay In Vitro

#### 2.2.1. Minimum Inhibitory Concentration (MIC) Assay

The model bacterial strains used in this work were *S. aureus*, *E. coli*, and methicillin-resistant *S. aureus* (MRSA). All bacteria were cultured in Luria–Bertani (LB) liquid culture medium (10 g/L tryptone, 5 g/L yeast extract, and 10 g/L sodium chloride; pH~7.4). LB solid culture medium was prepared by the addition of agar powder (1.5%, *w*/*v*) to the liquid culture medium. The normal saline used in this work was 0.9% sodium chloride aqueous solution.

Briefly, 50 μL of logarithmic bacterial suspension (10^6^ CFU/mL) and 50 μL of various doses of ACDs were mixed in a 96-well plate, and incubated in a 37 °C incubator for 24 h. The turbidity of bacterial suspensions at 600 nm (OD_600_) in wells was recorded to evaluate the antibacterial rate of ACDs using a SYNERGY H1 microplate reader (BioTek, Washington, DC, USA). The lowest concentration of ACDs in wells where no bacteria was observed was the MIC value.

#### 2.2.2. Spread Plate Method

Here, 1 mL bacterial suspensions (10^6^ CFU/mL) with various doses of ACDs (0, 1.25, 2.5, 5, 10 μg/mL) were incubated in a 37 °C incubator shaker at 180 rpm for 3 h. Then, the bacterial suspensions were diluted 1000 times, and 100 μL was taken out to spread onto LB agar plates. After that, the plates were incubated in the 37 °C incubator for 24 h or 72 h. And independent plates were used for each incubation period to validate the antibacterial effect of ACDs.

#### 2.2.3. Time-Dependent Kinetic Analysis

Here, 100 μL of logarithmic bacterial suspension (10^6^ CFU/mL) and 100 μL of various doses of ACDs were mixed in a 96-well plate and incubated in a 37 °C incubator for 72 h. The turbidity of the bacterial suspensions at 600 nm (OD_600_) in wells was recorded to evaluate the antibacterial rate of ACDs using a SYNERGY H1 microplate reader (BioTek, Washington, DC, USA).

### 2.3. Cellular Toxicity Test

Mouse fibroblast cells (L929) and human keratinocytes cells (Hacat) were purchased from procell (Wuhan, China) and beyotime (Shanghai, China), respectively. The cells were cultured in Dulbecco’s Modified Eagle’s Medium (DMEM) with 10% fetal bovine serum (FBS) and 1% penicillin-streptomycin solution. Cells were seeded in 96-well plates at a density of 5 × 10^3^ per well, and incubated at 37 °C to adhere. Then the medium was replaced with 100 µL of fresh DMEM containing different doses of ACDs, and re-incubated for another 12 h. After that, the cell viability was measured and calculated according to the instructions of the CCK8 kit, using a SYNERGY H1 microplate reader (BioTek, USA).

### 2.4. Antibacterial Mechanism Investigation for MRSA

#### 2.4.1. Bacterial Morphology Study

MRSA bacterial cells (10^8^ CFU/mL) were incubated with 30 µg/mL of ACDs in a 37 °C shaking incubator at 180 rpm for 6 h. Afterwards, the cells were collected by centrifugation (8000 rpm, 5 min) and fixed with 2.5% glutaraldehyde at 4 °C. After washing, dehydration, and gold-sputtering steps, bacterial morphology was captured using scanning electron microscopy (JEOL Jsm-it800, Tokyo, Japan). Bacteria treated with normal saline were used as the control group.

#### 2.4.2. Live/Dead Staining

Live/dead bacterial staining was used to evaluate the cell integrity of MRSA cells after the treatment of ACDs. First, 1 mL MRSA suspensions in normal saline (10^7^ CFU/mL) were incubated with 2.5 µg/mL of ACDs in a 37 °C shaking incubator at 180 rpm for 2 h. Afterwards, cells were collected by centrifugation (10,000 rpm, 5 min) and stained with a SYTO9/PI double staining kit. The visual assessment of live/dead bacterial cells was captured using confocal fluorescence microscopy (CLSM). Bacteria treated with normal saline served as the control group.

#### 2.4.3. Zeta Potential and Membrane Potential Measurement

Here, 1 mL MRSA suspensions (10^8^ CFU/mL) were incubated with ACDs (final concentration of ACDs: 40 µg/mL), and the ζ potential of the solution at 5 min, 0.5 h, and 4 h was recorded using a Malvern Nano ZS zetasizer (Malvern, UK), respectively. The ζ potentials of ACDs alone and MRSA cells alone were also measured. All the ζ potential assays mentioned above were conducted in a 0.9% sodium chloride aqueous solution (normal saline).

Rh123 dye was used for the measurement of the cell membrane potential of MRSA cells after the treatment of ACDs. Briefly, 1 mL MRSA suspensions in normal saline (10^7^ CFU/mL) were incubated with ACDs in a 37 °C shaking incubator at 180 rpm for 2 h. Bacterial sediments were collected and co-incubated with 1 µM of Rh123 for another 30 min. Fluorescence intensities of green emission in bacterial cells were recorded and analyzed using flow cytometry (Cytoflex S, Beckman Coulter, Brea, CA, USA). Bacterial cells treated with normal saline served as the control group.

#### 2.4.4. Membrane Permeability Measurement

Here, 1 mL MRSA suspensions in normal saline (10^7^ CFU/mL) were incubated with ACDs in a 37 °C shaking incubator at 180 rpm for 2 h. Bacterial sediments were collected. Afterwards, N-phenyl-1-naphthylamine (NPN, 1 µM) was co-incubated for another 30 min to measure the cell membrane permeability of MRSA cells. The fluorescence spectrum of the solutions, ranging from 360 nm to 600 nm excited by 350 nm, was recorded using an FS5 spectrophotometer (Edinburgh, UK). Bacterial cells treated with normal saline served as the control group.

#### 2.4.5. Intracellular ROS Detection

Bacterial cells of MRSA in normal saline (10^7^ CFU/mL) were pre-treated with 20 µM DCFH in a 37 °C shaking incubator at 180 rpm for 30 min. After that, the bacterial sediments were collected and washed with normal saline. The bacterial sediments were re-dispersed in normal saline and co-incubated with ACDs in a 37 °C shaking incubator at 180 rpm for 1.5 h. Afterwards, fluorescent intensities of green emission in bacterial cells were recorded and analyzed using flow cytometry (Cytoflex S, Beckman Coulter). Bacterial cells treated with normal saline were used as the control group.

#### 2.4.6. Nucleic Acid Concentration Determination

Bacterial suspensions of MRSA (10^7^ CFU/mL in normal saline) were co-incubated with ACDs for 6 h (final concentration of ACDs: 10, 40 µg/mL). Afterwards, the supernatant was collected by centrifugation (12,000 rpm, 5 min). The UV–vis spectrum and absorbance at 260 nm of the supernatant were recorded using Lambda (PerkinElmer, Waltham, MA, USA). Bacterial cells treated with normal saline served as the control group.

### 2.5. Statistical Analysis

All data were presented as the mean ± standard deviation (SD). Each experiment has repeated three times at least. Data were analyzed using Student’s *t*-test. ** *p* < 0.05 was considered statistically significant.

## 3. Results and Discussion

### 3.1. Synthesis and Characterization of ACDs

**Figure 1:**
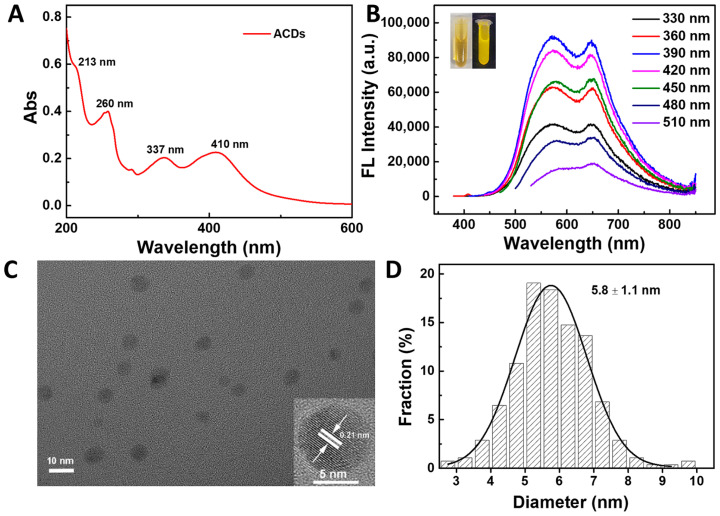
(**A**) UV–vis spectrum of ACDs; (**B**) fluorescent emission spectrum under different excitations; (**C**) TEM and high-resolution TEM images; (**D**) particle size distribution.

Herein, ACDs were synthesized using ascorbic acid and hexadecylpyridinium chloride monohydrate as precursors using a bottom-up approach (Scheme 1). As shown in Figure 1A, the UV–vis absorption spectra of the ACD solution showed two strong peaks at 213 nm and 260 nm, which were attributed to the π-π* transition of C=C in the conjugated aromatic sp^2^ structure [30]. The weak peaks at 337 nm and 410 nm are attributed to the n-π* transition of C=O and C-N bonds [31]. As shown in the inset of Figure 1B, the aqueous solution of ACDs exhibited a light-yellow transparent liquid in daylight, and emitted bright orange fluorescence when excited by 365 nm. The fluorescent emission spectra of ACDs under the various excitations are presented in Figure 1B. Notably, the fluorescent peaks of ACDs displayed no obvious change when the excitation ranged from 330 nm to 510 nm, indicating their excitation-independent fluorescent emission behavior (Figure 1B).

**Figure 2:**
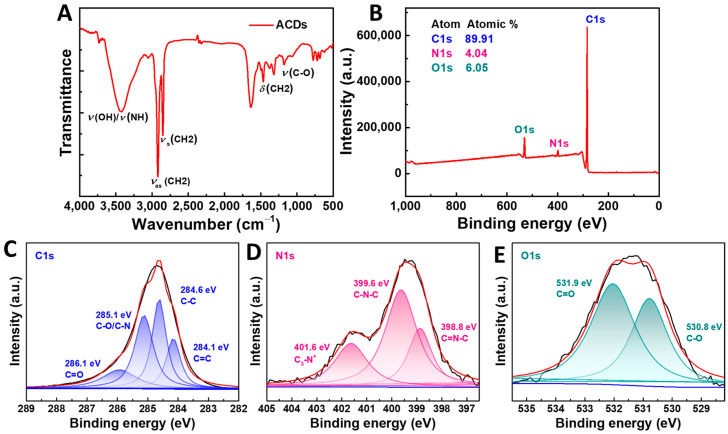
(**A**) FTIR spectrum of ACDs; (**B**–**E**) XPS spectra and high-resolution XPS spectra of C1s, N1s, and O1s.

The morphology of ACDs was characterized by TEM. As shown in Figure 1C, ACDs exhibited nanosphere-like structures, with an average diameter of 5.6 ± 1.1 nm (Figure 1D). It should be noted that clear lattice fringe spacings of 0.21 nm were observed in high-resolution TEM images of ACDs, which corresponds to the (100) plane of graphene (inset of Figure 1C) [32]. This indicates the good crystallization of the carbonic core of ACDs. The FTIR and XPS spectra of ACDs were collected and are shown in Figure 2. In the FTIR spectrum, broad absorption at 3115–3649 cm^−1^ was attributed to the stretching vibrations of the O-H/N-H bond [33]. The strong absorption peaks at 2923 cm^−1^ and 2848 cm^−1^ could have been contributed by asymmetric and symmetric stretching vibrations of CH_2_, while the absorption peak at 1463 cm^−1^ was due to the bending vibration of CH_2_ [34,35,36]. These results clearly confirm the existence of massive -OH/NH_2_ and long-alkyl-chain functional groups on the surfaces of ACDs. The full-scan spectrum of XPS in Figure 2B indicates that the main elements making up ACDs were carbon, nitrogen, and oxygen, with atomic percentages of 89.91%, 4.04%, and 6.05%, respectively. The high-resolution spectra of C1s showed four peaks at 284.1 eV (C=C, graphitic carbon), 284.6 eV (C–C, graphitic carbon), 285.1 eV (C–O/C–N, sp^3^ carbon), and 286.1 eV (C–O, sp3 carbon), indicating that ACDs were rich in oxygen-containing groups on their surfaces (Figure 2C) [37]. There were three peaks at 398.8 eV (C=N–C), 399.6 eV (C–N–C), and 401.6 eV (C_3_-N^+^) in the high-resolution spectrum of N1s (Figure 2D) [38,39]. In the high-resolution spectrum of N1s, two peaks at 530.8 eV and 531.9 eV can be ascribed to C–O and C=O bonds, respectively (Figure 2E). These results indicate that ACDs are rich in nitrogen-containing surface functional groups, making them efficient in antibacterial applications.

### 3.2. Antibacterial Activity of ACDs

Before the antibacterial assay of ACDs, the cell viability of mouse fibroblasts cells (L929) and human keratinocytes cells (Hacat) with the co-incubated ACDs was tested to evaluate their biosafety. As shown in Figure S1, with the concentration of ACDs increasing from 0 to 10 µg/mL, the cell viability of L929 cells was maintained at >80% and the cell viability of Hacat cells was maintained at >75%. These results indicate that the obtained ACDs had relatively low cytotoxicity.

**Figure 3:**
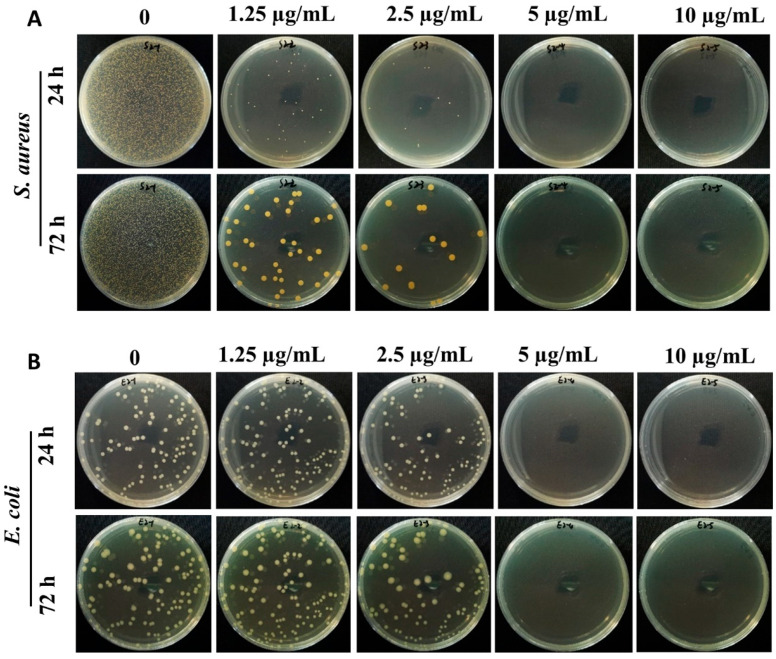
Antibacterial activity of ACDs against *S. aureus* (**A**) and *E. coli* (**B**). Typical photos of bacteria after 24 h and 72 h of treatment with different concentrations of ACDs on LB agar medium.

The antibacterial activity of ACDs was evaluated using the MIC test and spread plate method. The MIC test showed that the prepared ACDs had excellent inhibitory effect against the growth of *S. aureus*, *E. coli*, and MRSA. The MIC values of ACDs towards *S. aureus*, *E. coli*, and MRSA were 2.5, 5, and 2.5 µg/mL. As shown in Figure 3, after incubation in a 37 °C incubator for 24 h, large amounts of bacterial colonies were observed in the untreated control groups. In contrast, the numbers of bacterial colonies significantly reduced when the concentrations of ACDs increased from 1.25 to 10 µg/mL. Interestingly, when the co-incubated time was extended to 72 h, the inhibited effects of ACDs against *S. aureus* and *E. coli* persisted.

**Figure 4:**
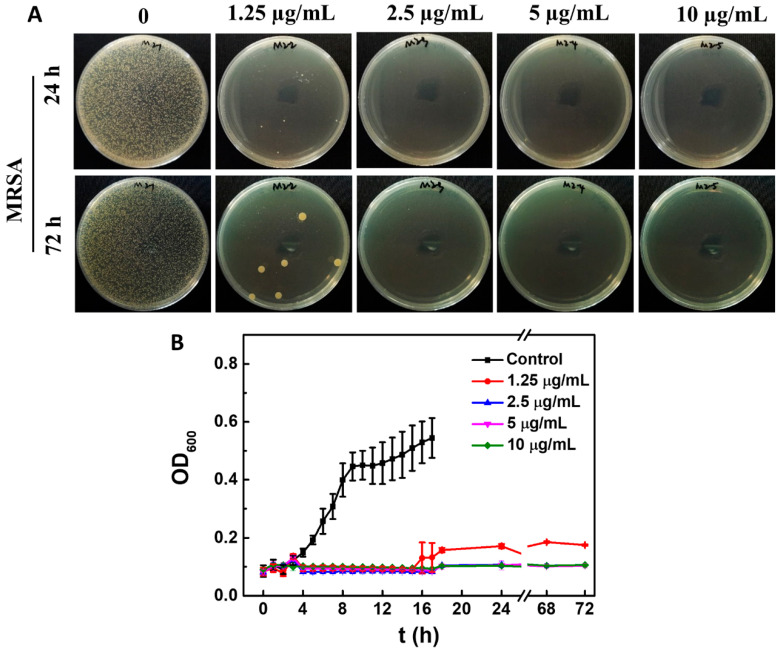
Antibacterial activity of ACDs against MRSA. (**A**) Typical photos of MRSA after 24 h and 72 h of treatment with different concentrations of ACDs on LB agar medium; (**B**) growth curve of MRSA cells with or without ACDs.

In addition, highly effective inhibitory effects of ACDs were also observed on drug-resistant bacteria. Similarly, 2.5 µg/mL of ACDs could completely inhibit the growth of MRSA for as long as 72 h (Figure 4A). Furthermore, the growth curve of MRSA cells with ACDs over 72 h was measured. As shown in Figure 4B, the optical density at 600 nm (OD_600_) of the untreated bacterial group increased rapidly within the first 24 h. In contrast, the OD_600_ values of MRSA bacteria with ACDs remained at around 0.1 when the concentrations of ACDs varied from 1.25 to 10 µg/mL, indicating the excellent antibacterial activity of ACDs. These results indicate that ACDs exhibit a highly efficient and long-term inhibitory effect towards bacteria, and especially drug-resistant bacteria.

Different CDs and their antibacterial effects from the previous literature are summarized in Table 1. As shown in Table 1, the synthesis method used in this work was more eco-friendly, time-saving, and relatively safe. Importantly, compared with the CDs in the table, the obtained ACDs exhibited lower MIC values against *S. aureus*, *E. coli*, and MRSA bacteria, and inhibited the growth of MRSA bacteria over a long period, indicating the higher performance of antibacterial effects and better long-term antibacterial activity of ACDs. In addition, these results proved our hypothesis that using precursors containing pyridine and long alkyl chains to prepare CDs would greatly enhance the antibacterial activity of the target product, which was consistent with the results reported previously [35,40].

### 3.3. Mechanism of Antibacterial Activity

To reveal the underlying antibacterial mechanism, a comprehensive analysis of bacterial cells was conducted, including changes in cell morphology, cell integrity, cell membrane potential, and cell membrane permeability, as well as intracellular reactive oxygen species and nucleic acid release.

**Figure 5:**
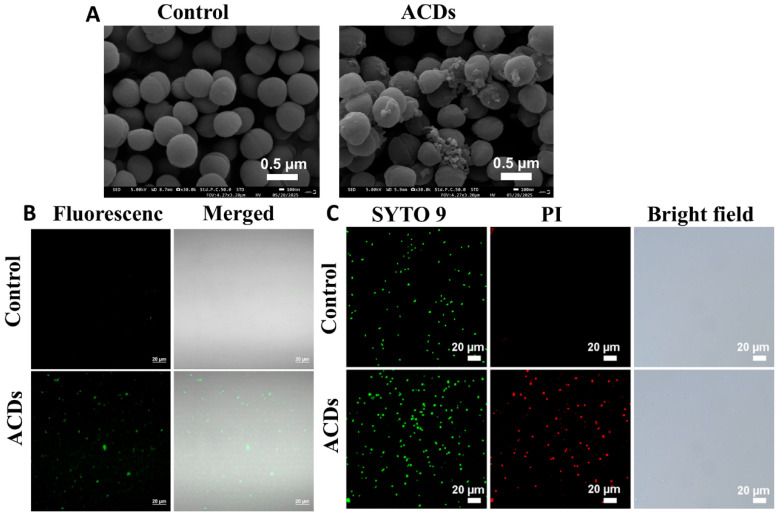
(**A**) SEM images of MRSA cells treated with normal saline or ACDs; (**B**) bacterial imaging of ACDs excited by 405 nm laser; (**C**) fluorescent images of MRSA cells stained with SYTO 9 and PI, respectively.

SEM was used to characterized the morphology after the treatment of ACDs. As shown in Figure 5A, the control group MRSA cells were rounded, complete, and had smooth surfaces. However, after the treatment with ACDs, the morphology of MRSA cells showed wrinkles, breakage, and leakage of intracellular contents. By leveraging the orange fluorescence emission of ACDs, the internalization of ACDs inside the MRSA bacteria was studied. As shown in Figure 5B, bright fluorescence was observed in the bacteria cells with the incubation of ACDs at 2.5 µg/mL for 2 h, whereas there was no obvious fluorescence in the untreated bacterial cells. These visual images confirmed the internalization of ACDs inside the MRSA bacteria. Next, the destructive effects of ACDs on MRSA bacteria were studied by SYTO 9/propidium iodide (PI) double-staining experiments. All bacteria could be labeled with SYTO 9 with green fluorescence, whereas the bacteria with ruptured cell membranes could be highlighted by PI, which emits enhanced red fluorescence when binding to intracellular nucleic acid [44]. The analysis results of the live/dead bacterial staining are shown in Figure 5C. Bacteria in the control group exhibited green fluorescence, confirming the cell integrity of normal bacteria. In contrast, bright red fluorescence was observed in bacteria treated with ACDs, indicating that serious disruption of the cell integrity occurred after the treatment with ACDs. The live/dead staining results indicate that the treatment of ACDs could cause severe cell damage and reduce bacterial survival rates, which is consistent with the SEM characterization.

**Figure 6:**
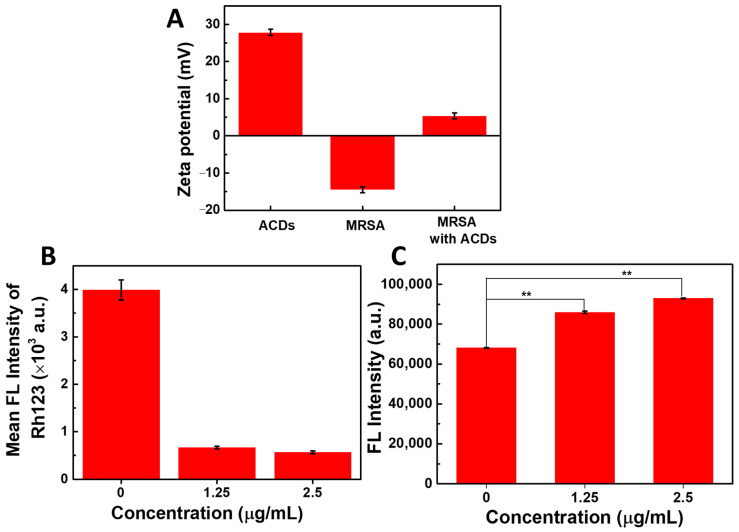
(**A**) Zeta potential of ACDs and MRSA bacteria; (**B**) quantitative analysis of cell membrane potential by flow cytometry; (**C**) fluorescence change in NPN dyes after co-incubation of MRSA bacteria with ACDs. ** represents *p* < 0.05.

As shown in Figure 6A, the surface potentials of ACDs and MRSA cells were measured as +27.86 mV and −14.50 mV, indicating that there might be strong electrostatic interactions between ACDs and bacteria. This was confirmed by the positive potential of MRSA cells treated with ACDs (+5.39 mV). To acquire more insights into the dynamic behavior of ACDs when in contact with bacterial surfaces, the potential of MRSA cells treated with ACDs was recorded at different time intervals. As shown in Figure S2A, the zeta potential of the MRSA cells remained positive when the incubation time increased from 5 min to 4 h, which suggested that the electrostatic interactions were stable over time. Additionally, the colloidal stability of ACDs was evaluated by the fluorescent spectrum (Figure S2B). It can be concluded that the fluorescence emission peaks and intensity of ACDs in normal saline exhibited neglect changes compared with those in water, indicating the good stability of ACDs.

Following the co-incubation of bacteria with ACDs, a rhodamine 123 (Rh 123) probe was used to examine the cell membrane potential; it could emit green fluorescence in normal bacteria, and almost no fluorescence in bacteria that risked membrane potential collapse. As presented in Figure 6B, quantitative analysis results of flow cytometry showed that green fluorescent intensity in MRSA cells decreased by nearly 75% after the treatment of ACDs (2.5 µg/mL), indicating the collapse of bacterial membrane potential.

Next, cell permeability changes in MRSA bacteria with or without ACDs were examined. N-phenyl-1-naphthylamine (NPN, 1 µM) is a hydrophobic label agent that exhibits weak fluorescence in water, and can emit enhanced fluorescence when entering a hydrophobic environment. For normal bacterial cells, NPN molecules were hindered in the culture medium by the complete cell wall and cell membrane. However, once the cell wall/membrane were disrupted, amounts of NPN molecules could enter the bacteria and emit bright fluorescence. As presented in Figure 6C, compared with the control group, the fluorescence intensity of NPN in bacteria that were co-incubated with ACDs greatly increased. The fluorescence intensity increased by 126% at 1.25 µg/mL of ACDs, and 136% at 2.5 µg/mL of ACDs, indicating the increase in bacterial permeability.

**Figure 7:**
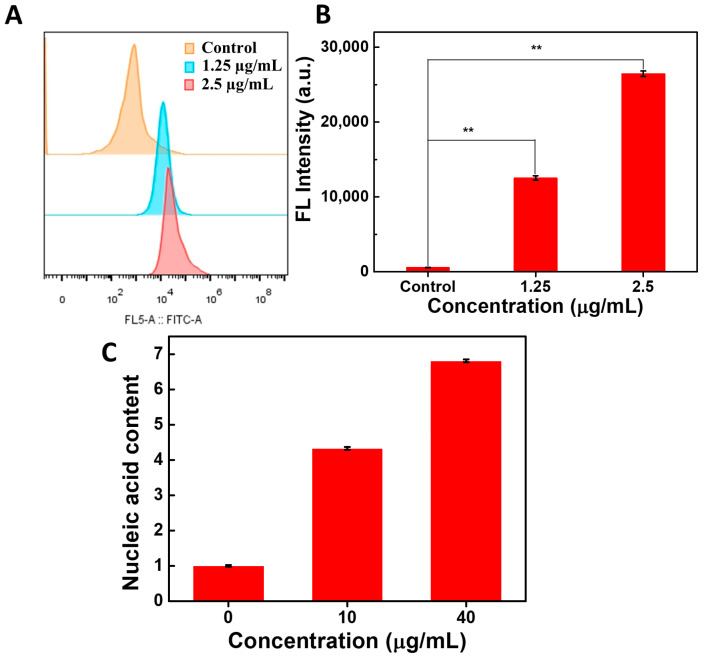
Fluorescence detection of DCF by flow cytometry (**A**) and the quantitative analysis results (**B**); (**C**) the amount of bacterial nucleic acid leakage after the co-incubation of MRSA bacteria with ACDs. ** represents *p* < 0.05.

Subsequently, the generation of ROS in MRSA bacteria was measured using a dichlorofluorescin diacetate (DCFH) probe, which could emit bright green fluorescence after the oxidation of ROS [45]. As shown in Figure 7A, compared with the control group, the fluorescent intensity of DCF in bacteria that were co-incubated with ACDs greatly increased. Quantitative analysis results of flow cytometry showed that compared with the control group, the green fluorescence intensity of DCF increased more than 21-fold when the concentration of ACDs was 1.25 µg/mL, while it increased more than 45-fold when the concentration of ACDs was at 2.5 µg/mL of ACDs. These results indicate that a large amount of ROS was produced within the bacterial cells (Figure 7B).

Furthermore, the concentration of leaked nucleic acids in the bacterial supernatant was quantified. The nucleic acid concentration in the bacterial supernatant that was co-incubated without ACDs was set as the control. As shown in Figure 7C, compared with the control group, a 4-fold concentration of leaked nucleic acids was observed in the bacterial supernatant that was co-incubated with ACDs. These results were consistent with those observed using SEM. It can be concluded that ACDs exerted significant destructive effects on bacteria by causing damage to bacterial structures and leakage of internal nucleic acids.

Taken together, these findings indicate that the obtained ACDs have high efficiency and a long-term inhibitory effect against MRSA bacteria. It can be concluded that antibacterial effects were initially achieved by disrupting the cell wall/membrane structure through the insertion of long alkyl chains and strong electrostatic interactions between bacteria and ACDs. Next, the collapse of cell membrane potential and the increase in bacterial permeability allowed amounts of ACDs to enter. After penetration into the bacteria, ACDs induced cellular oxidative stress via the production of excessive ROS, which might further destroy the cell wall/membrane, causing a leakage of nucleic acids and other content, ultimately leading to cell death.

## 4. Conclusions

In this work, antibacterial ACDs were synthesized via a bottom-up approach. Quaternary ammonium function groups containing long alkyl chains were successfully grafted onto the surface of CDs during their synthesis process. The obtained ACDs exhibited a highly inhibitory effect against MRSA bacteria with minimum inhibitory concentrations at 2.5 µg/mL. Crucially, 2.5 µg/mL of ACDs could inhibit the growth of MRSA for as long as 72 h, which has rarely been reported. Antibacterial mechanism studies revealed that ACDs killed the MRSA bacteria mainly through disrupting the cell wall/membrane, destroying cell membrane potential, inducing the generation of reactive oxygen species, and causing a leakage of nucleic acids and other intracellular contents. In sum, our work provided a kind of ACD with high efficiency and long-term antibacterial activity, offering promising potential for combating drug-resistant bacterial infections. In addition, it should be noted that there are several limitations of this work. For instance, the environmental stability of ACDs needs to be studied further. The impact of ACDs on the evolution of bacterial resistance is also worthy of evolution. Efforts in mouse models of bacterial infection in vivo will help expand the application of the obtained ACDs. These are also the directions of our future work.

## Figures and Tables

**Table 1:** Summary of different CDs and their antibacterial effects.

CD Label	Synthesis Process	MIC Values	Long-Term Antibacterial Activity	Ref.
Q-CQDs	Curcumin and GTA reacted in autoclave at 200 °C for 24 h	4, 8, 8, and 32 µg/mL against *S. episermidis*, *S. aureus*, MRSA, and *E. coli*	Not mentioned	[41]
Cur-CDs	Curcumin and GTA reacted in autoclave at 200 °C for 8 h	40, 20, 20, and 60 µg/mL against *E. coli*, *P. aeruginosa*, *S. aureus*, and MRSA	Not mentioned	[21]
GCDs	Onion powder reacted in autoclave at 210 °C for 4 h, and reacted at 170 °C for 40 min with PHMB	3.75, 5, 5, and 7.5 µg/mL for *B. subtilis*, MRSA, *S. aureus*, and *E. coli*	Not mentioned	[42]
QCDs	CA and DDA reacted in an oil bath at 150 °C for 210 min	30, 15, and 15 µg/mL against *E. coli*, *S. aureus*, and MRSA	Not mentioned	[43]
G-CDs	CA and DDA reacted in an oil bath at 150 °C for 2.5 h, and at 170 °C for 1 h with PHMG	5, 5, 10, and 10 µg/mL against *S. aureus*, MRSA, *E. coli*, and *S. marcescent*	MIC values against *E. coli*, *S. aureus*, and MRSA for 48 h	[36]
ACDs	CPC and AA reacted in an oil bath at 80 °C for 1 h	2.5, 5, and 2.5 µg/mL against *S. aureus*, *E. coli*, and MRSA	2.5 µg/mL against MRSA for 72 h	This work

## Data Availability

Data will be made available on request.

## Supplementary Materials

The following supporting information can be downloaded at https://www.mdpi.com/article/10.3390/nano15171296/s1, Figure S1. Cellular toxicity of ACDs on mouse fibroblasts cells (L929) and human keratinocytes cells (Hacat). Figure S2. (A) zeta potential of MRSA cells after the treatment of ACDs (final concentration: 40 µg/mL); (B) the fluorescence spectrum of ACDs in water and normal saline.
