# Supplementary material for: High Efficiency and Long-Term Antibacterial Carbon Dots for Combating Antibiotic Resistance
Source: Nanomaterials (Basel). 2025 Aug 22;15(17):1296. doi: 10.3390/nano15171296 (PMC12430245; doi:10.3390/nano15171296)
Supplement: Supplementary file 1 [file nanomaterials-15-01296-s001.zip › nanomaterials-3815785-supplementary.pdf]

Supplementary material

High efficiency and long-term antibacterial carbon dots for combating antibiotic resistance

Beibei Wang [a], Dandan Zhang [a], Gang Zhou [a], Xiaodong Li [b], Tingli Sun [a], Qingshan Shi [a], Xiaobao Xie [a, *]

[a] Guangdong Provincial Key Laboratory of Microbial Culture Collection and Application, State Key Laboratory of Applied Microbiology Southern China, Institute of Microbiology, Guangdong Academy of Sciences, Guangzhou 510070, China

[b] School of Life Sciences, Guangzhou University, Guangzhou, Guangdong 510006, China.

[*] Corresponding author: Xiaobao Xie

[*] E-mail addresses: xiexb@gdim.cn (X. Xie)

Table of contents in Supplementary material

1. Cellular toxicity of ACDs on mouse fibroblasts cells (L929) and human keratinocytes cells (Hacat) (Fig. S1).
2. (A) zeta potential of MRSA cells after the treatment of ACDs (final concentration: 40 μg/mL); (B) the fluorescence spectrum of ACDs in water and normal saline (Fig. S2).

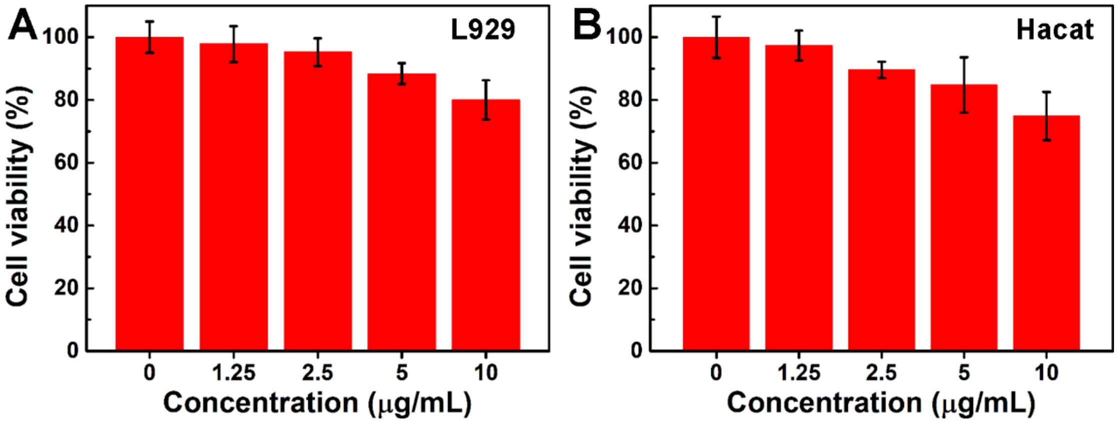

Fig. S1 Cellular toxicity of ACDs on mouse fibroblasts cells (L929) and human keratinocytes cells (Hacat).

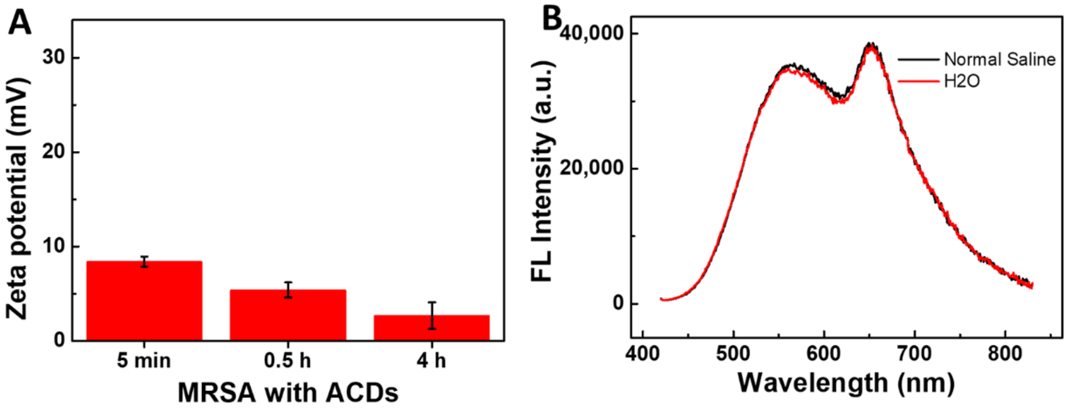

Fig. S2 (A) zeta potential of MRSA cells after the treatment of ACDs (final concentration: 40 μg/mL); (B) the fluorescence spectrum of ACDs in water and normal saline.
